# Letter to the Editor: Percutaneous closure of an iatrogenic common femoral arteriovenous fistula secondary to extracorporeal membrane oxygenation access: a case report

**DOI:** 10.1186/s42155-026-00683-x

**Published:** 2026-04-09

**Authors:** Hiroki Kuji, Naoki Hayakawa, Hiromi Miwa, Yasuyuki Tsuchida, Shinya Ichihara, Satoshi Hirano, Kotaro Miyaji, Shunichi Kushida

**Affiliations:** Department of Cardiovascular Medicine, Asahi General Hospital, Chiba, Japan

**Keywords:** Iatrogenic femoral arteriovenous fistula, Perclose ProGlide, Endovascular therapy

We report a case of successful percutaneous common femoral arteriovenous fistula (AVF) closure using a Perclose ProGlide closure device (Abbott Vascular).

A 56-year-old man who was receiving treatment for acute myocardial infarction decannulated the venoarterial extracorporeal membrane oxygenation (VA-ECMO) cannula inserted into the right common femoral artery (CFA) and common femoral vein (CFV) percutaneously (Fig. 1A) [1]. Although VA-ECMO decannulation and hemostasis were successful, post-decannulation angiography revealed a common femoral AVF (Fig. 1B, C). The fistula had a short and wide neck. In most cases, if AVF is not leading to high-output cardiac failure in a critically ill patient, then common practice is close observation and follow-up and/or open repair. However, due to concern of potential deleterious hemodynamic effects of high-output cardiac failure after discontinuation of ECMO in a patient who has recently suffered an acute myocardial infarction, we elected to perform urgent closure of AVF. We accessed the AVF directly with a 21-G needle (Merit Advance angiography needle; Merit Medical) with the overlay image of the angiogram. A 0.014-inch guidewire (Gladius MGES guidewire; Asahi Intec) was inserted via the needle, and the guidewire was advanced into the right superficial femoral artery (Fig. 1D). A Corsair Armet microcatheter (Asahi Intecc) was inserted into the inner cylinder of the 6-Fr sheath to reduce the gap between the inner sheath and the 0.014-inch wire [2]. The 6-Fr sheath was inserted, and the 0.014-inch wire was replaced with a 0.035-inch Radifocus wire, and we inserted the Perclose ProGlide device (Fig. 1E). We confirmed the presence of a footplate on the arterial wall by ultrasound and performed suturing AVF on the arterial wall side (Fig. 2). Then balloon angioplasty of CFA and CFV were performed with Senri 8.0/40 mm (TERUMO, Tokyo, Japan) and Mustang https://doi.org/10.0/40 mm (Boston Scientific, Marlborough, MA, USA) balloons respectively (Fig. 1F). Angiography confirmed the complete occlusion of AVF (Fig. 1G). Subsequently there was a complete resolution of AVF on follow-up ultrasound which was performed 6 months later.

**Fig. 1:**
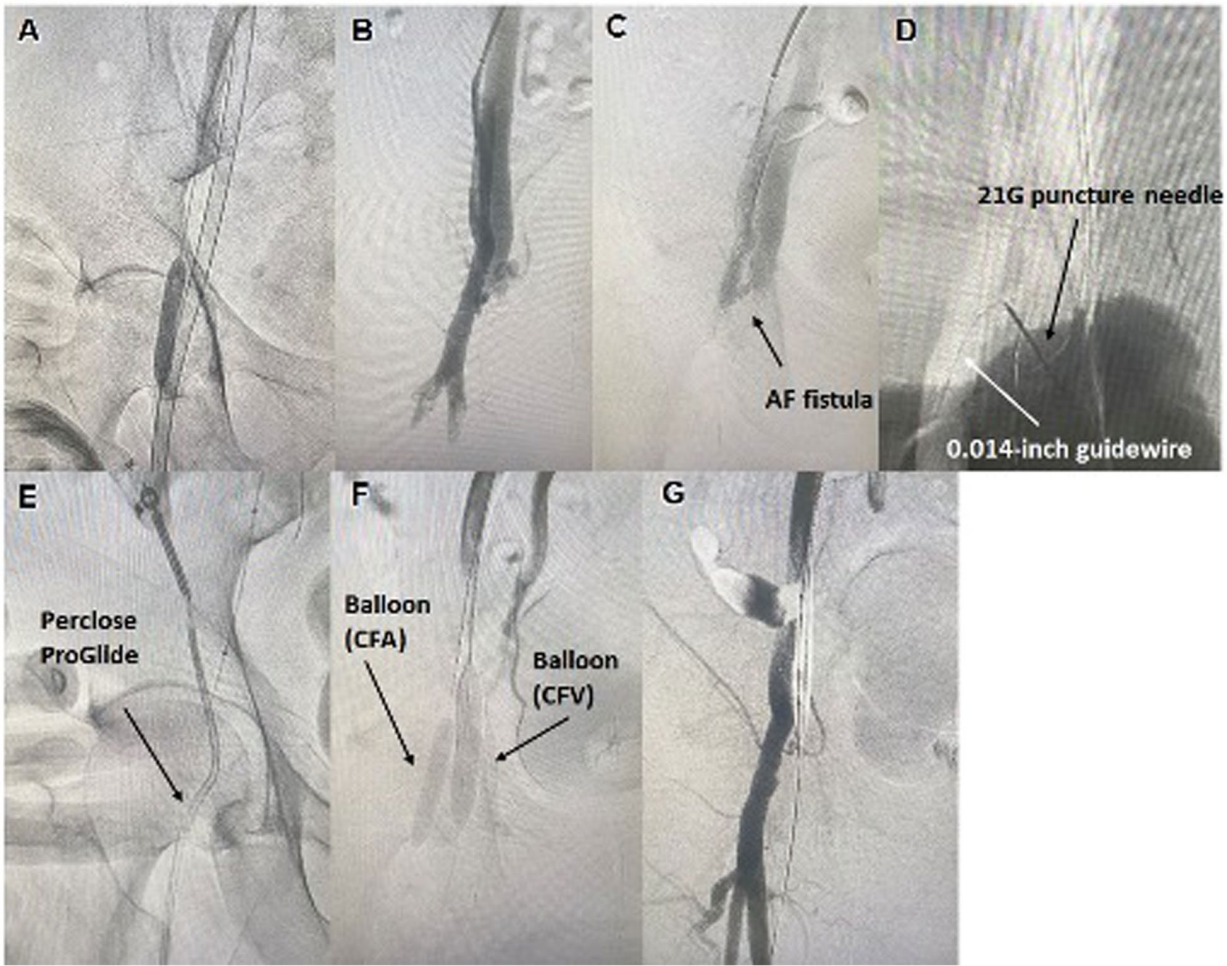
Angiographic imaging. **A** Demonstrating successful VA-ECMO CFA access removal after successful balloon occlusion of right common iliac artery via a left radial approach guiding sheath (not shown in this image). This was followed by balloon angioplasty of right CFA at the ECMO access site for hemostasis (shown). **B**, **C** Digital subtraction angiography in two different projections demonstrating absence of extravasation at the ECMO access site confirming complete hemostasis and the presence of AVF (black arrow). **D** Percutaneous puncture of AVF with 21 gauge needle (black arrow) under fluoroscopic guidance, followed by advancement of 0.014-inch guidewire (white arrow) into the right superficial femoral artery. **E** Demonstrating advancement of Perclose ProGlide closure device via AVF into SFA over a 0.035-inch Radifocus wire for deployment and closure of SFA arterial wall defect and AVF. **F** Demonstrating balloon angioplasty of CFA and CFV at the site of AVF after deployment of Perclose ProGlide closure device. **G** Final angiography demonstrating complete occlusion of AVF and patent CFA

**Fig. 2:**
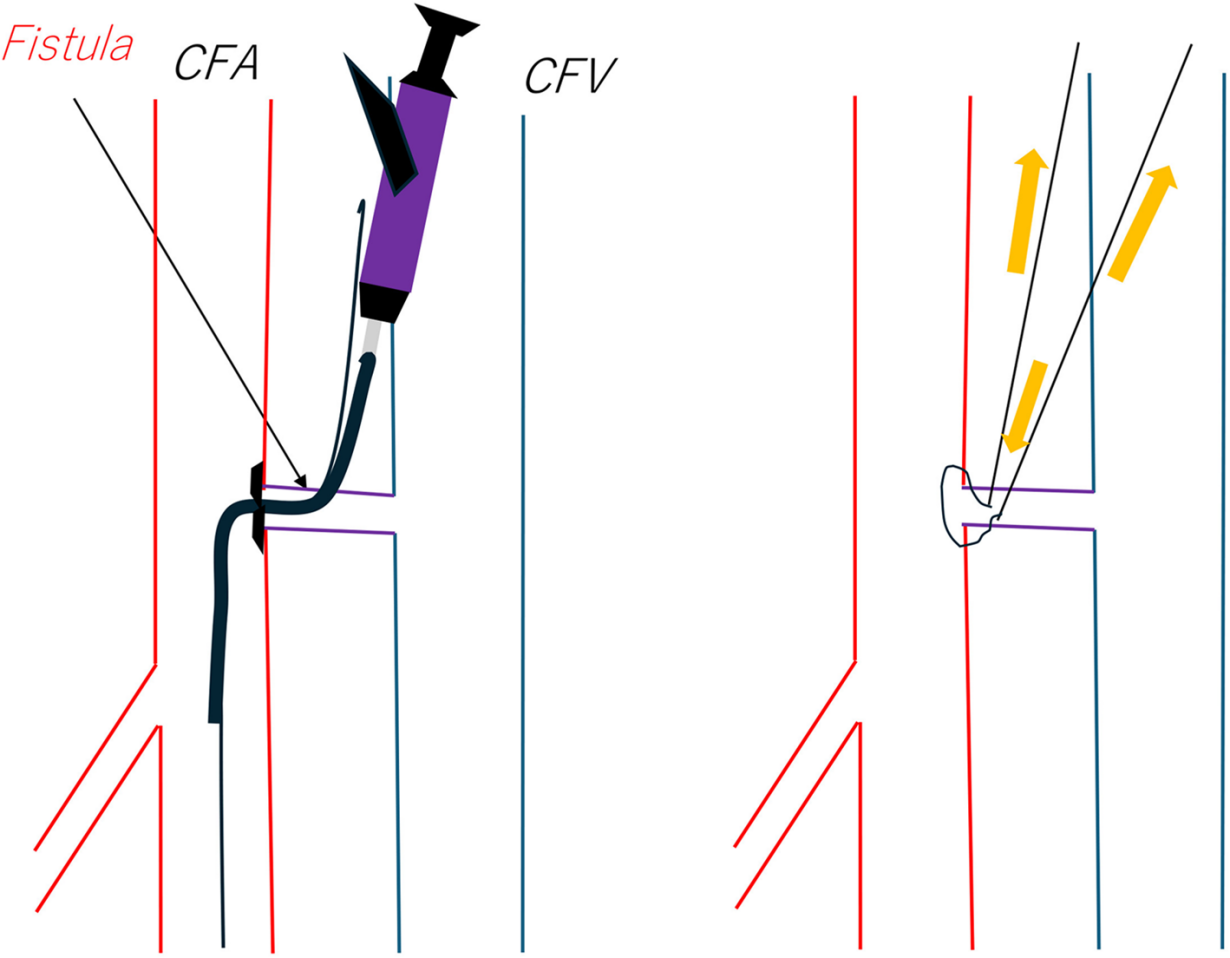
Diagrams demonstrating optimal location of footplate and how to suture arterial wall defect and AVF using a Perclose ProGlide

The standard management of common femoral artery AVF is an open surgical repair. It has a risk of complications such as groin infection, bleeding, and lymphorrhagia [3], and dual antiplatelet therapy can be a relative contraindication. Stent-graft placement in the CFA should be avoided because of the risk of deep femoral artery occlusion, compression or kinking, and the disadvantage of limiting the access route for catheterization [4].

Although there are no prior reports describing the use of Perclose ProGlide device for the closure of AVF, similar techniques have been reported for the treatment of pseudoaneurysms using this device [2, 5]. In these cases, the pseudoaneurysm was directly punctured, and a guidewire was passed through the neck into the artery, where it was sutured on the arterial wall side successfully. This approach requires a wire and closure device to traverse the neck. In our case, the fistula was wide and clearly visible on angiography, so we were able to treat it by directly accessing the fistula and inserting the Perclose ProGlide into the artery. This technique may be less suitable for the closure of AVF with a long and tortuous neck. In such cases, embolization can be an appropriate option.

This case was concluded with no complications. If Perclose ProGlide fails to suture the arterial wall defect, then it is necessary to consider alternative options such as open repair or coil embolization. The possible complications are arterial or venous obstruction, or bleeding. In such cases, open surgical repair may be required. This technique when used under careful sonographic guidance may offer a feasible treatment option for iatrogenic AVFs, particularly those with a short and wide neck or involving the CFA. Further investigations and more data are warranted to evaluate the safety and effectiveness of this technique.

## Data Availability

The datasets generated and/or analyzed during the current study are not publicly available because individual privacy could be compromised but are available from the corresponding author upon reasonable request.

## Abbreviations

AVF: Arteriovenous fistula
CFA: Common femoral artery
CFV: Common femoral vein
EVT: Endovascular therapy
VA-ECMO: Venoarterial extracorporeal membrane oxygenation
